# Empowering professionals: An intensive short course on fundamentals of clinical data science

**DOI:** 10.1017/cts.2025.10170

**Published:** 2025-10-10

**Authors:** Richard F. Ittenbach, Brian McCourt, Maurizio Macaluso

**Affiliations:** 1 Division of Biostatistics and Epidemiology, Cincinnati Children’s Hospital, University of Cincinnati College of Medicine, Cincinnati, OH, USA; 2 Duke Clinical Research Institute, Duke University, Durham, NC, USA

**Keywords:** Clinical data science, clinical data management, curriculum, data science, executive training

## Abstract

Clinical data science, like the broader discipline of all data science, has quickly grown from obscurity only a few decades ago to one of the fastest growing specialties in biomedical research today. Yet, the education and training of the workforce has not kept pace with the growth of the field, the complexity of science, or the needs of the profession. The purpose of this paper is to provide a template for an intensive short course on fundamentals of clinical data science that meets the needs of working professionals in academic, industry, and government research settings. Care will be taken to introduce students to essential roles, responsibilities, and practice patterns within the field, the foundational components from which they come, and many of the soft skills needed for professional practice and advancement in the field today. The course is designed as an evidence-based, immersive learning experience taught over a 5-day period on a university campus, taught using principles of best educational practice and multiple modalities, to assure optimal interaction and engagement throughout the week. This template may be reproduced by any institution interested in and capable of offering such a program.

## Introduction

Clinical data science, like the broader discipline of all data science, has quickly grown from obscurity only a few decades ago to one of the fastest growing specialties in biomedical research today [1]. Yet, the education and training of the workforce has not kept pace with the growth of the field, the complexity of the science, or the needs of the profession. A new approach to education is desperately needed for the field, and its dedicated professionals, to reach their full potential today [2].

The pace, complexity, and sophistication of clinical research continues to expand well beyond what was imagined only a few decades ago [3, 4]. With a typical Phase III clinical trial now having 263 procedures, 22 endpoints, and 3.6M data points (up from 7 endpoints, 100 procedures, and 0.5M data points in 2005) [5] and with 60% of clinical sites now using more than 20 overlapping software applications at any one time [6], even the more rigorously trained professionals find it challenging to keep up. Concomitant rises in ethical issues, cost of healthcare, and advancing technologies have raised the stakes even further, necessitating that today’s clinical data scientists’ understanding goes well beyond simply managing the data to now include the usability of the data, their merits as a scientific tool, and their role in the scientific process [7–9]. The scientific community is now expecting these professionals to be true “scientists.”

While there are many programs training data scientists, there are currently no programs training “clinical” data scientists – at either the graduate or undergraduate levels. Today’s clinical data scientists have generally received their training through a collection of efforts and opportunities [2]. The most rigorous cases are traditional or rebranded statistics or informatics programs, which are strong with respect to analytics, systems development, and technology, but often lack formal training in the data itself and most certainly lack the ability to manage the flow of data through a clinical research study. The more typical cases are employer-driven continuing education programs focusing on the processing of data rather than the theory, rationale, or scientific attributes of the data. Professional associations have offered specialized courses and programs just as individuals have created unique individually-tailored programs; however, these are not reliable mechanisms to train an entire workforce.

A trend that has become apparent in the business community over the past several decades is to bring working professionals together for targeted, brief, intensive, immersive educational experiences [10]. Such courses, often referred to as executive education courses, have made in-roads in the business world and traditional university communities, alike [11–13]. According to Mena-Guacas et al., short courses offer benefits not typically available through other instructional formats, such as being taught by professional educators used to delivering the content, relocating students from the typical office and work-day routines to a more typical learning environment, and a holistic integration of content [14]. The benefits of using an evidence-based framework add to the impact of the experience through an enriched appreciation of content, higher levels of trust in the instructors, and instructors’ ability to package the information in a more usable format for learners [11, 15].

As with all investments, efficacy and potential for a return on one’s investment remains a concern. Beginning with the managerial revolution of business schools in the United States and Harvard Business School’s Advanced Management Program as a model for other programs in 1945, programs are now taught worldwide across a wide range of formats [16, 17]. In a survey of 52 corporations conducted by the Consortium for University-based executive education programs, 92% of companies reported using participant feedback data sources to evaluate their executive education programs and 49% use it in career tracking and promotions, with 60% connecting executive education to their overall strategy [18].

Given the pace and complexity of clinical research today, the lack of instructional programs globally, and the general lack of educational opportunities for working professionals, the purpose of this paper is to provide a template for an intensive short course on fundamentals of clinical data science that meets the needs of working professionals in academic, industry, and government research settings.

## Materials and methods

### Expectations of the course

**Figure 1.**
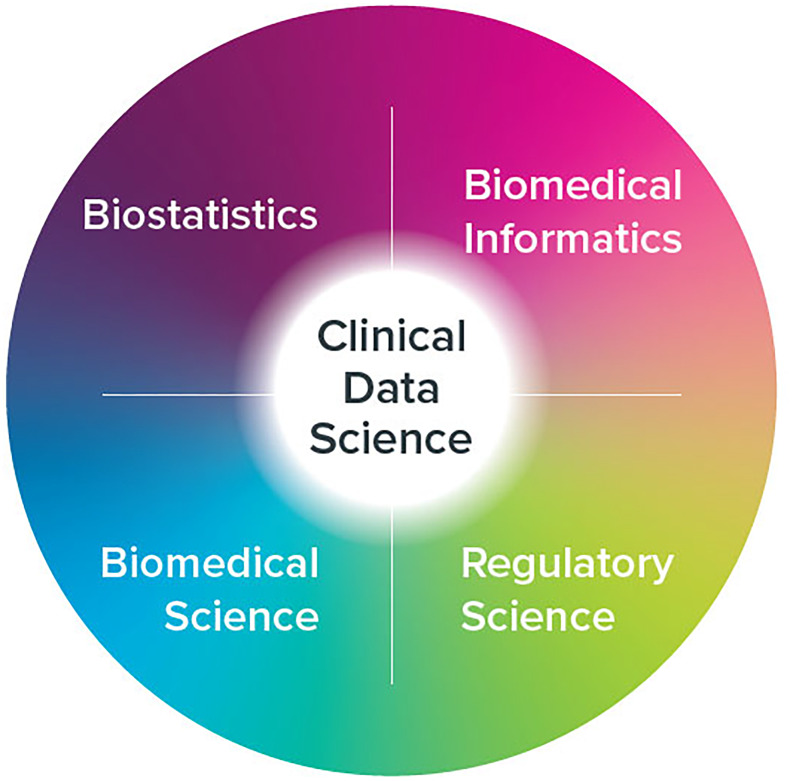
Clinical data science components.

This week-long course was developed with several expectations in mind: first, to map the coursework onto existing curricula and competency frameworks in the professional literature, specifically drawing from the foundational domains of biostatistics, biomedical informatics, biomedical science, regulatory science, and the clinical research literature, more generally (see Figure 1) [8, 19–24]. The course, while short, needs to be a snapshot of content shared in a graduate level program in clinical data science “*devoted to the measurement, acquisition, care, treatment, and inferencing of clinical research data.”* Second, the content must be scaled to the abbreviated nature of the program and the accelerated pace of the instruction, but still prioritize weaving the information into a coherent whole, with each module building on the ones that came before it and laying the foundation for the ones that come after it (vertical articulation) [25]. Third, for it to truly be a clinical data science course, it needs to show fidelity to “science” and to the scientific method. Furthermore, executive education faculty are encouraged to incorporate experiential learning activities to better integrate knowledge with applications of the material in typical work settings.

As with all scientific disciplines, there is core content to help define the discipline and supporting content that connects the new knowledge base to the other sciences. In the case of clinical data science, what began as an operational subspeciality to help get drugs and devices to market faster and with better reliability, effectiveness, and safety has evolved into a scientific specialty in its own right, one that draws from the scientific knowledge bases of related, foundational disciplines [26, 27]. Consequently, a short course in clinical data science should consist of core knowledge representing the essential components of the field such as first principles, roles and responsibilities, and fundamentals of design and implementation, while also orienting learners to core tenets of the disciplines on which the new field is based (biostatistics, biomedical informatics, biomedical science, and regulatory science) [2].

### Pedagogy

Implicit within the course is a commitment to adhering to principles of best educational practice. That is, to meet the students where they are professionally, to engage them using multiple instructional formats (lecture, case study, group discussion), and to introduce them to exemplary models of literature and professional practice. While all instructional modules are organized around specific learning objectives, not all learning will come from structured exercises. Some will come from the less structured parts of the course by design – the breaks, meals, and networking. The course should offer an educational experience that is greater than the sum of its parts and stimulate learning and development once the students leave the course.

To be consistent with the educational characteristics of a graduate program, this intensive, abbreviated course strives to relay content from a full graduate program, just in a more condensed form. If the course is successful, it will move the students toward a deeper understanding of clinical data science. To help guide the curriculum and provide a point of reference for what students will be learning in the short course, we offer seven learning objectives – one from each of our three core modules and one from each of the four foundational disciplines of biostatistics, biomedical informatics, biomedical science, and regulatory science.

With respect to the core modules, the learning objectives begin with mapping a very simple data-flow diagram and process onto the scientific method (Objective 1), to recalling from memory and describing elements of a data management plan (Objective 2), to actually connecting data fields and variables from the study protocol to the statistical analysis plan, to the data management plan – and, then finally, to the study database (Objective 3). Implicit in these core modules is a developmental sequence that takes the learner from the scientific method to the plan for the data to how the fields and variables progress through all phases of the study from both an operational and a scientific standpoint.

**Figure 2.**
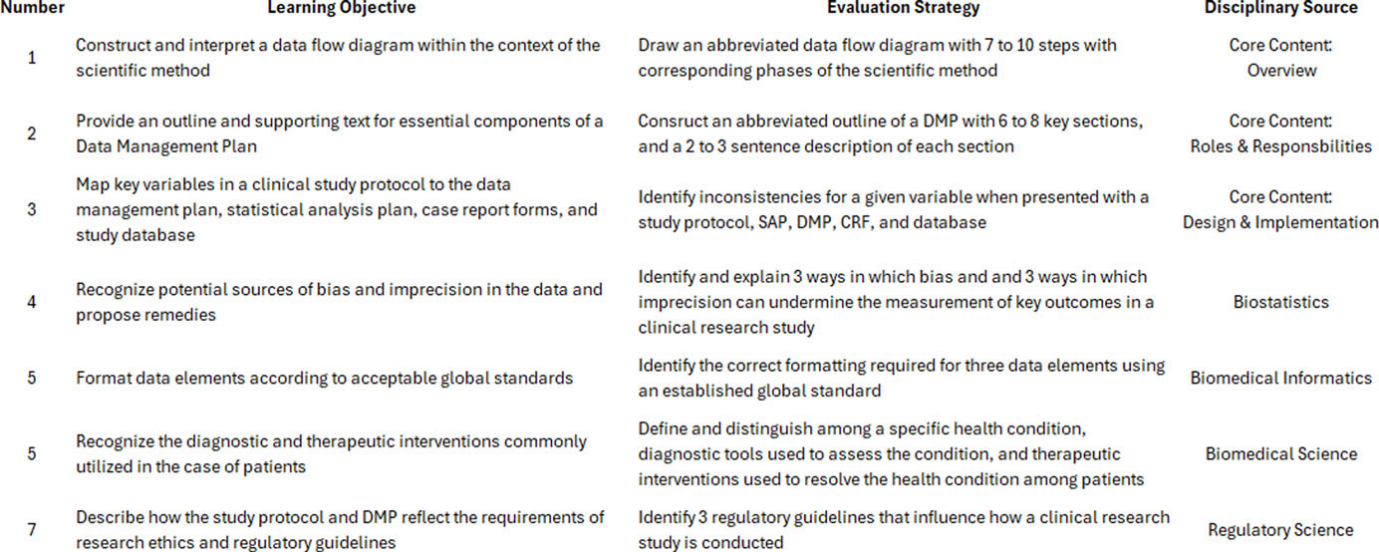
Short course learning objectives and evaluation.

Regarding the research courses that support the core content, the learner moves from a recognition and understanding of the impact of measurement bias and imprecision on the scientific process (Objective 4, biostatistics); to formatting data elements using globally-recognized standards (Objective 5, biomedical informatics); to distinguishing among various health conditions of patients and research participants along with the role of diagnosis and intervention in responding to those conditions (Objective 6, biomedical science); and, finally, an understanding and reliance of influential guidelines protecting human subjects in clinical research (Objective 7, regulatory science). Please see Figure 2 for a list of the seven learning objectives for this short course.

As important as the roadmap for learning is, the educational process would not be complete without an evaluation plan. As such, the evaluation mechanism used for the short course will be similar to the one used for our graduate program and includes a two-phase approach to program evaluation. Phase I will include a straightforward pretest-posttest approach to evaluation in which the students are asked a series of questions related to typical work products, immediately prior to and immediately following delivery of the content (see Figure 2 for examples of the work product evaluation). In each case, the assessments will be designed to be succinct, focused, time limited, and based on content used in the course. Phase II will consist of components from the Environment, Pedagogy, Institution, and Course (EPIC) system, which is a standardized (NSF funded) evaluation system of surveys for university-level data science programs – but tailored to our short course rather than that of an entire semester-long course [28].

One challenge will be to find a meaningful but efficient way of incorporating homework into the intensive short course. One strategy that is available to instructors is to have the students bring specific examples of work products with them to the short course, that they can use in the break-out sessions following content-related discussions (e.g., Data Management Plan, abbreviated protocol, data-flow diagram). Foundational concepts can be effectively demonstrated using data science tools that faculty employ in real-world applications. With the widespread availability of online data, online data-collection methods, databases, and analytical tools, these elements can be integrated into case studies, enabling participants to gain a deeper understanding of the material encountered in traditional classroom discussions. Finally, the course must be developed in keeping within the fiscal expectations and limitations of host institutions.

### Budgetary considerations

As with all innovative programs, this one must be funded in a way that is not only affordable but is also sustainable and does not deplete resources from other programs and initiatives. As such, we have provided a preliminary budget that details typical costs of such a program (see Table 1).

**Table 1.** Example budget

Cost category	Cost	Cost with 20% service fee	Cost with 20% fee, 7.5% tax
Facilities			
Marketing and materials	2,500		2,688
1 Breakout room ($800/day)	4,000	4,800	5,160
AV, internet, onsite support staff ($3,000/day)	15,000		16,125
Facilities total			23,973
Catering (meals, snacks, reception)			
Breakfast ($25 × 5 days × 50 participants [10 staff])	6,250	7,500	8,063
Lunch ($35 × 5 days × 50 participants [10 staff])	8,750	10,500	11,288
Breaks ($30 × 25 participants × 9 break periods)	6,750	8,100	8,708
Dinners ($50 × 3 days × 55 participants [15 staff])	8,250	9,900	10,643
Alcohol ($10/glass × 50 participants × 2 glasses)	1,000	1,200	1,290
Catering total			39,992
Speaker travel, honoraria			
Honoraria ($3,500 × 10 faculty, $500 × 10 faculty)	40,000		40,000
Travel and expenses for speakers ($1,000 × 7 faculty)	7,000		7,000
Honoraria and Travel Total			47,000
Project management support			
Project manager (2080 hrs x ½ yr × 0.30 effort x $100/hr)	31,200		31,200
Total			142,165

*Note*. catering costs for instructional faculty and staff should be built into the overhead costs.

The proposed budget is divided into four categories: facilities, catering, speaker travel/honoraria, and project management support. As one might imagine, implementing an intensive, 5-day course such as this one requires meticulous planning and coordinated efforts as well as time for follow-up communication and post-course processing. The higher the quality, the more likely students are to attend from great distances, and, as a result, the greater the need for communication, planning, and organizational support. We have proposed the use of a skilled project manager at 30% time for six months. The underpinning value of this program lies in the carefully curated and coordinated content and opportunities for interaction with leaders in the field. Unlike traditional university courses based on a single instructor, a multidisciplinary program such as this one requires the input and synthesis of multiple experts from many areas, driving a significant portion of the budget. Facilities and catering are the most easily quantifiable, and the most easily scaled to align with the expectations and needs of the program. Cost estimates provided in Table 1 are drawn from actual examples of conferences offered at two university medical centers and experienced with on- as well as off-campus continuing education courses and meetings.

## Results

The clinical data science course is designed to be a 50-hour course consisting of 19 educational modules taught over an intensive, five-day period. The 19 modules are organized as follows:

- 5 primary modules associated with core content (overview, roles and responsibilities, design and implementation, field placements, good clinical data management practice)
- 5 supporting modules associated with foundational research content (biomedical informatics, biostatistics, biomedical science, regulatory science, and clinical research ethics)
- 4 supporting modules on soft skills needed for successful professional practice (leadership, critical thinking, team science, communication)
- 5 supporting modules devoted to challenging cases studies.

Day 1 of the program begins with an opening session, which includes a light breakfast, an introduction to the program, faculty, course objectives, housekeeping, and news and notes for the day. The remaining days will begin similarly but be much more focused, beginning with a light breakfast, a recap of the prior day, an introduction to the current day and any news or notes that may be needed. The instructional portion of the day is divided into morning and afternoon sessions, with each half-day beginning with a substantive content session (e.g., Overview of Clinical Data Science) followed by a supporting case study or instruction in a “soft skill” deemed important for professional practice (e.g., critical thinking). A number of different instructional methods will be used to keep delivery fresh and of interest to students with different areas of interest, ranging from the traditional didactic method to hands-on exercises and small group discussions.

**Figure 3.**
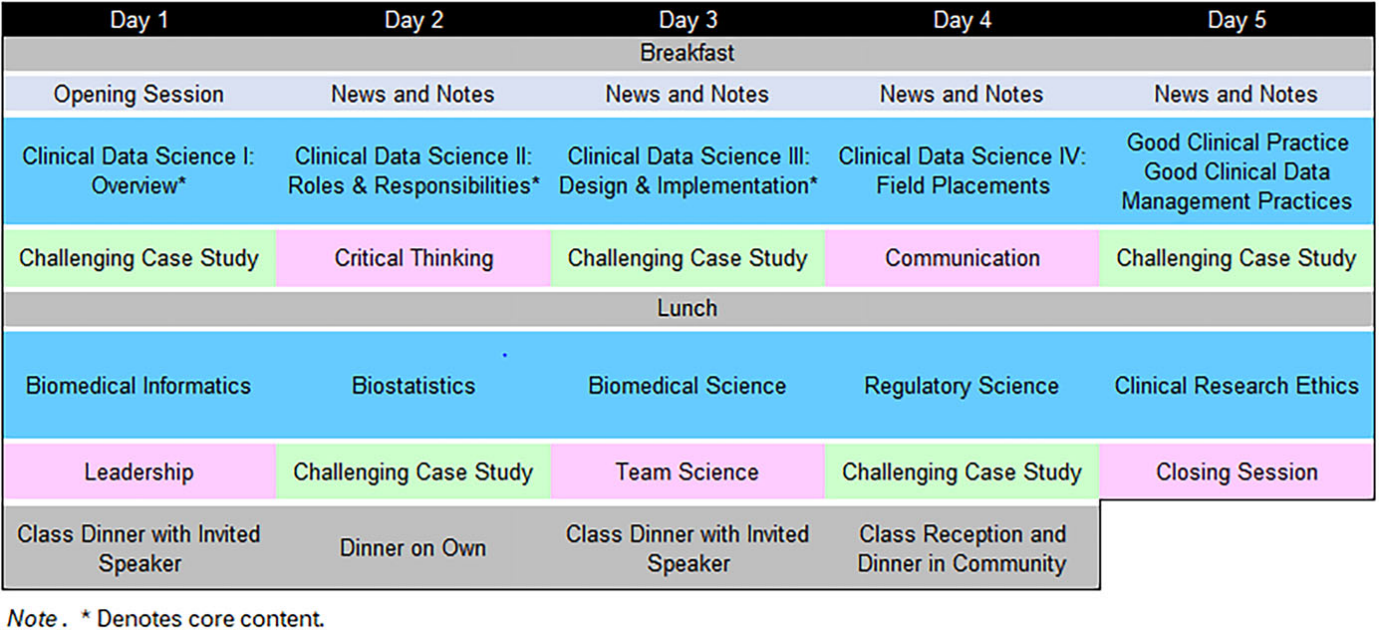
Example curriculum.

When people are brought to campus for an intensive and demanding short course, especially when brought to campus from great distances, having meals available and aligned with the conference are crucial to keeping the students present, focused, and on schedule. As alluded to previously, even the meals are designed to offer the students time to network with faculty, staff, and other students – to be fun and relaxing but still immerse them in the field of clinical data science. Three of the five days will end with a working dinner and a professional speaker from the community (see Figure 3).

The most beneficial component of a short, intensive program such as this one is what it can do for current, working professionals who may not have the time or resources to enroll in a more traditional, multiyear program. While the benefits of the course will be shaped by the instructors as much as each student’s commitment to learning, this short course will be designed to equip the students with an integrated knowledge base that extends the foundational content of the parent disciplines. Provided below are examples of ways in which the short course will benefit working professionals, organized by the four respective foundational areas:

### Biostatistics

Data science as a distinct discipline is rooted in statistics (or biostatistics) and computer science. As such, the new discipline draws heavily from the analytical side of clinical inquiry – one that is premised upon searching for certainty in a world of unknown, but probabilistic events. Appreciation of the uncertainty that necessarily affects data capture and the importance of a rigorous statistical approach to harnessing the uncertainty of the inferences and the point estimates on which the estimates are based will require a critical understanding of study design and the ways in which the data are measured and collected. The connection among fundamentals of measurement, the statistical tests used to analyze the data, and other supporting principles discussed in most introductory statistics courses will highlight the importance of the rationale, validity, and need for the precision of the biomedical sciences. Such relationships are generally detailed in the core content, Statistical Analysis Plan, and its companion document, Data Management Plan, noted above (Objectives 2, 4).

### Biomedical informatics

Information science and the technology that supports it provides the means to ensure scientific rigor to a degree previously deemed unachievable – across all areas of science. Mastery of the data capture software and the programming techniques that support it may allow presenting survey questions with branching logic in a user-friendly format, representing the data entry modules in a table that reproduces a table of events, displaying summary tables reporting which forms have been completed or how many data elements are missing. Alignment of data formats to existing standards will ensure that the data produced are “findable, accessible, interoperable and reusable” (FAIR), enhancing the impact of the clinical research study. The Clinical Data Interchange Standards Consortium (CDISC) has developed standards that cover most aspects of clinical research, from protocol development to data collection, presentation, and analysis. Adherence to CDISC standards is mandatory for submission of data to the U.S. Food and Drug Administration (FDA) (Objectives 3, 5).

### Biomedical science

Understanding the research questions that motivate a clinical study, the students will verify how the study protocol operationalizes the scientific objectives into a schedule of events and outlines the procedures needed to capture interventions and endpoints in a synthetic study flow diagram. It is also important, however, for the clinical data scientist have some basis for understanding the health condition being studied, diagnostic strategies used, and avenues of intervention. The mechanism through which the health-related data will be collected, processed, and stored should be specified in a data management plan [29] and its corresponding manual of operations, the roles of key clinical research staff, who in turn will interface with the clinical data managers (Objectives 3, 6).

### Regulatory science

The technical aspects of designing and deploying a clinical research study must be embedded in an overarching ethical/legal framework that reinforces the principles of respect for persons, beneficence, and distributive justice specified in the Belmont Report and further developed in the current body of rules and regulations governing research with human subjects [30]. Understanding these principles is indispensable for clinical data scientists, whose technical expertise must be put at the service of the ethical treatment of research participants. Thus, the research protocol must not only reflect the scientific objectives of the study but must align the study procedures and objectives in such a way that review boards can make an independent assessment of the risks and benefits to the participants. The work of the data management team must be inspired by the respect for the autonomy and privacy of the participants and must adhere to all applicable data confidentiality regulations (Objectives 1, 7).

Integration of principles and concepts from the four foundational areas of biostatistics, biomedical informatics, biomedical science, and regulatory science transforms a clinical data manager into a “clinical” data scientist [31, 32]. A clinical data scientist does not simply use the data as a tool but rather treats the clinical research data as a scientific object in its own right, worthy of study, and further scientific development. The clinical data scientist strengthens the precision of the data to achieve the objectives of a research study and enhances the usability of the data and its power to better serve clinical and translational science, in general [4].

## Discussion

Executive education courses offer a new and potentially exciting option for filling the gap in continuing education opportunities for working professionals. Extensive short courses such as the one presented here can delve more deeply into selected domains and still put the information into a broader context. Executive education programs remain a vastly under-utilized form of continuing education fully capable of having an impact at both the individual and institutional levels [12]. This paper provides a template for an intensive short course on fundamentals of clinical data science that meets the needs of working professionals in academic, industry, and government research settings.

The most notable feature of this course is instruction in the core tenets of clinical data science – the measurement, acquisition, care, treatment, and inferencing of clinical research data [2]. The data are not simply the product of research but the basis for it. This is the material that distinguishes the content from other content received in other settings. It is the material that distinguishes this profession from all others. With respect to the core content noted previously, following is a snapshot of the content covered during the morning sessions:

- Overview of the field, focusing on the data as the basis for scientific hypotheses and the inferences that come from them: Module 1
- Roles and responsibilities of today’s data scientists: Module 2
- Design and implementation, from development of the protocol to database lock and closeout, and every step in between: Module 3
- Simulation of field-based work: Module 4
- Good Clinical Practice/Data Management Practice Guidelines: Module 5

All of the above are critical components of a clinical data scientists’ world and routines. Modules 1–5 will be the featured content in the mornings, respectively (see Figure 3).

### Research courses

The field of clinical data science is little more than 50 years old. Yet, the field as it is known today actually evolved out of other related fields, initially at the intersection of statistics and computer science, but now with heavy influence from the biomedical and regulatory sciences. The fact that today’s professionals must now deal with complex issues not fully appreciated in years past, requires that today’s clinical data scientists receive instruction in the tenets of clinical research ethics. Whereas core content will be featured in the morning, with the research courses featured in the afternoons:

- Principles of biomedical informatics, the linking of computational tools and algorithms with biomedical information and data: Module 6.
- Principles of biostatistics, inferencing, and clinical trial design: Module 7
- Principles of biomedical science, including that which directly relates to healthcare: Module 8
- Federal regulatory guidelines and principles that shape biomedical research today, particularly those that pertain to the protection of research subjects: Module 9
- Clinical research ethics, a topic that is often passed over for the more familiar and comfortable analytical content: Module 10.

### Supplemental courses

Recognizing that science is no longer practiced in a vacuum, scientists are now expected to work closely and collaboratively with investigators from multiple disciplines. Consequently, the closing hour of each morning and afternoon will be devoted to either a challenging case study reinforcing principles presented in that day’s session, or supplemental content on one of four soft skills deemed critical to the practice of clinical data science and advancement in the field: critical thinking, communication with colleagues, leadership fundamentals and, finally, team science skills (Modules 11 through 14), with challenging case studies designed to illustrate the challenges in today’s intricate and fast-paced world of biomedical research (Modules 15 through 19). Sessions featuring soft skills and challenging case studies are designed to be offered in alternating sessions (see Figure 3).

### Benefits of short courses

For individuals whose training has become outdated, those trained in a different area of expertise, or even those whose supervisors simply want the staff member to have additional training, intensive short courses can provide the education needed for strengthening one’s professional practice. Whereas it is often difficult to devote time to a semester-long course that may be disruptive to a person’s life or work schedule, most colleagues understand dedicating several days to continuing education. And most will agree that 40 to 50 hours of formal instruction by university faculty is substantial enough to improve one’s knowledge base irrespective of level. Whether through supplemental readings, repetition of concepts, increased exposure time, or in-depth dialog between the students and instructors, intensive short courses are likely to produce returns on investment well beyond what employer- and learner-driven webinars can typically offer.

Not surprisingly, students are not the only ones who benefit from these short, intensive courses. Courses such as these can also have tremendous appeal for university faculty. Staffing the courses with experienced university faculty who are familiar with the content makes sense from an administrative standpoint. They know the material and the pitfalls – and, in many cases, have dealt with the barriers to practice. This is their world. What university faculty have not always encountered, however, are the problems of managers and employees in the trenches. Here faculty get to see the problems and pitfalls of practice through a different lens, by seasoned administrators and employees, which can be both exciting and challenging for all involved. Because the courses are short, university faculty are often willing to invest their time in the area and with students who have an acute need to know. The faculty can then return to their normal schedules. In short, university faculty can often draw from both theory and practice to go deeper, quicker than others for whom the teaching is an add-on to their normal work routine.

In addition, universities often appreciate the inclusion of short courses to their portfolio of classes. These courses often bring non-traditional students to campus, expanding the exposure of the program and the university to new groups of potentially interested students. In addition, these courses have the potential to put university faculty and staff in direct contact with industry professionals, opening opportunities for other forms of collaboration, consultation, and engagement. And why not expand the portfolio of learning opportunities to these new groups of students? The best data available suggest that universities currently account for less than 1% of the executive education market – a market that universities are most qualified to compete in – and, one for which there is ample room for growth in this profoundly important area of professional development [10, 33]. Intensive short courses and the opportunities for interaction mean that industry professionals also have access to new faculty with whom they can interact with when needed.

As noted previously, faculty should strive to incorporate a number of different instructional models in their teaching. As working professionals know, professional education classes can often be long and boring. But, lessons learned from the best elementary and secondary teachers suggest that active engagement is the best way to learn and retain information. For this reason, it is recommended that short course faculty balance traditional lectures with small group discussions, as well as some activity-based assignments to allow the students to shape the direction of the content as well as be actively engaged with the material. An example of an activity-based assignment is to create a data-flow diagram, data-collection form, example code, or output using public datasets and freely accessible tools. This blend will likely need to be adjusted frequently based on the faculty’s strengths and the needs of the content. Asking students to bring examples from their prior work experience into activities can be a highly valuable way of engaging students and alignment to individual learning goals.

### Budgetary considerations

Education costs. More importantly, though, investments in education represent the values of a person, the community, and the profession. Intensive short courses such as the one presented here begin to make the content available to a much wider audience than ever before, working professionals from a broad range of backgrounds. The week-long commitment requires time away from family, work, and typical routines, but elevates the knowledge and skills of those who engage.

The course brings together and packages information that is not otherwise available to the professional community who can directly benefit. If the students leave the course being able to achieve the objectives listed here and begin operating at levels substantially above their previous levels of performance, everyone will benefit – the student, the studies they are working on, the employees they supervise and work with, the organizations in which they work, and science more generally. Investment in education pays dividends well beyond the cost of the instruction. But education does indeed cost; the more essential the education, the greater the cost. This program is an example. Organizations committed to these processes, and their outcomes may replicate this model, its purpose, and scope.

The budget provided in Table 1 is generated from conference expense sheets designed to increase professional education in biomedical research at the authors’ institutions. We are now applying that model to clinical data science. The short course presented here proposes a budget of approximately $140,000, which not all organizations will be able or even interested in offering. It is not a formal prescription for a one-size-fits-all course but may be scaled up or down to meet the needs of the students, sponsors, and institutions involved. There are always ways to trim expenses, and to scale the information to the budgets and resources available, either by using more local, less experienced instructors, offering the program in less expensive venues, and/or not providing snacks and meals. A piece-meal, fragmented system of webinars and short courses has been used with this segment of the workforce for decades, it is now time to offer them more. This is a profession that oversees increasingly complex biomedical data without any formal training – it is time to change that model – and offer them more training that is commensurate with their importance to the field.

No revenue projections were provided to allow institutions to gauge their own rates of return due to differences in what they are willing and able to offer. The intensive short course presented here is designed for industry-leading organizations and workforces willing to invest in training at the highest levels. For purposes of completeness, a registration fee of $3,500 for 40 students would cover the costs of the course described here, while a registration fee of $4,370 for the same 40 students would return a 25% margin for the host institutions.

Additional factors will also need to be considered such as student versus practicing professional rates as well as early bird and/or regular registration rates. Even deadlines for drop-outs can impact one’s budget markedly after expenses have been committed, so establishing firm drop-out deadlines can provide a cushion should drop-outs come too late for others to register. In addition, host institutions will need to decide if they wish to pursue sponsorship from educational and/or commercial organizations and whether they will want to offer continuing education credit. Such factors are usually institution-specific and were not factored into this model. As with all continuing education, not all organizations will be able or interested in undertaking this type of training for their community. This entire model can be scaled based on the needs of an organization.

### Evaluation

Care should be taken to evaluate not only the delivery of information (pedagogy) but the course’s timeliness and receptivity by the students to keep the material fresh and useful for the learners. The specifics of the evaluation tool should be up to the course organizers; however, one key component should be whether the students can accurately and effectively master the seven objectives presented in Figure 2. Other process-oriented questions on planning such as content, meals, and overall costs may be included, as well. The evaluation plan described here should be designed a priori, prior to the launching of the program, to help in determining its effectiveness and planning for subsequent instruction [34].

## Conclusion

The purpose of this paper was to provide a template for an intensive short course on fundamentals of clinical data science that meets the needs of working professionals in academic, industry, and government research settings. The 50-hour, 19 module course is divided into three sets of instructional modules: core content, supporting research content, and supplemental modules. Whereas the first set of modules is designed to instruct on the core content of the profession, the research modules are designed to convey information that serves as the basis for the new field, and, finally, the supplemental modules are designed to guide instruction on challenging case studies as well as many of the soft skills needed for practice as a clinical data scientist today.
